# Community-Based Intervention to Improve the Well-Being of Children Left Behind by Migrant Parents in Rural China

**DOI:** 10.3390/ijerph17197218

**Published:** 2020-10-02

**Authors:** Minmin Jiang, Lu Li, Wei Xing Zhu, Therese Hesketh

**Affiliations:** 1 Department of Social Medicine, Zhejiang University School of Medicine, Hangzhou 310058, China; 0015695@zju.edu.cn (M.J.); lilu@zju.edu.cn (L.L.); zhuweixing@hmc.edu.cn (W.X.Z.); 2 Centre for Global Health, Zhejiang University School of Medicine, Hangzhou 310058, China; 3 UCL Institute for Global Health, 30 Guilford Str., London WC1N1EH, UK

**Keywords:** community-based intervention, left-behind children, rural children, China, psychosocial well-being, school performance, Strengths and Difficulties Questionnaire (SDQ), internalizing problems, externalizing problems

## Abstract

In rural China around 60 million left-behind children (LBC) experience prolonged separation from migrant worker parents. They are vulnerable to a range of psychosocial problems. The aim of this study was to determine whether a community-based intervention consisting of Children’s Centres can improve psychosocial well-being and school performance of these children. The intervention was carried out in 20 villages, for children aged 7 to 15 years, irrespective of left-behind status. Nine hundred and twenty children, 438 LBC and 256 children living with parents (RC) attended the Centres. At follow-up after one year, there were improvements compared to baseline in total difficulties (measured with the Strengths and Difficulties Questionnaire) in children left behind by both parents (*p* = 0.009), children left behind by one parent (*p* = 0.008) and RC (*p* = 0.05). Postintervention school performance significantly improved in both categories of LBC (*p* < 0.001), but not RC (*p* = 0.07); social support score increased in both categories of LBC (*p* < 0.001) and RC (*p* = 0.01). Findings from interviews with key stakeholders were overwhelmingly positive about the impacts. With strong local leadership and community motivation, a low-cost intervention can improve children’s psychosocial well-being in these settings. Allowing communities to adapt the model to their own situation fosters local ownership, commitment, with benefits for children, parents, carers, and communities.

## 1. Introduction

In recent decades, migration has increased markedly in many parts of the world, with profound impacts for family structures and the home communities of migrants [1]. The primary driver of migration is the desire for improved socioeconomic circumstances and opportunities [2], but it often leads to separation between family members, leading to particular concerns about children separated from migrant parents for prolonged periods, often many years [3]. The widely-adopted definition for these so-called “left-behind children” (LBC) is children under 18 separated from one or both parents for at least six months. Using this definition, global estimates run to the hundreds of millions, accounting for around 40% of children in rural South Africa, 36% in Ecuador, and 27% in the Philippines [4]. In China, LBC accounted for an estimated 60 million or 36% of all rural Chinese children in 2017 [5].

Children’s wellbeing is influenced by a range of factors, so the effects of parental absence are complex. Parental migration affects child wellbeing through a trade-off between increased family income and disrupted parental care [6]. There is now a large literature about the impacts of separation from migrant parents on the health and well-being of children. Much of it emanates from China. A recent systematic review found that left-behind children had increased risk of depression, anxiety, and conduct disorder, compared with children living with parents [4]. They are also reported to have lower academic attainment [3], and higher risk of both unintentional injury [7], and abuse [8]. In China, extreme events such as rape, murder, and suicide among LBC have also been widely reported in the media [9].

Concerns for the welfare of these children have raised awareness about specific support to address their needs [10]. In 2013, the Chinese government called for rural communities to take responsibility for the education, care and protection of LBC [11]. There is very little literature describing interventions to improve their well-being [12]. The few studies from China are small-scale individual psychological counselling projects and family, group or school-based interventions [13,14,15,16,17,18]. Studies have suggested that group interventions can be effective in relieving anxiety and loneliness [14], as well as in enhancing resilience and subjective well-being of LBC [15,16]. A school-based intervention in a rural boarding school improved academic performance of LBC. There has been criticism of interventions being ineffective because they are ‘enforced’ by higher authorities [17].

An important exception is a community-based intervention conducted in two Chinese provinces [17]. This showed improvements in resilience, physical health and academic performance in 7–18 year olds, illustrating the potential effectiveness of community approaches. Community-based interventions present an obvious approach for addressing the needs of left-behind and other vulnerable children, given that these children mainly live in villages, which, in China, typically consist of small well-organised communities, with clear leadership. Community-based interventions generally combine individual and community strategies, aiming to promote well-being among population groups in a defined local community [19]. They have proven effective in improving outcomes in children across a range of difficult circumstances and settings [20].

The primary aim of our study was to establish whether a community-based intervention could improve psychosocial well-being and academic performance of LBC and rural children living with parents (RC). (While our original proposal was aimed at LBC, a preliminary survey, showed that there were small and inconsistent differences between LBC and rural children across a range of well-being measures, so to carry out an intervention to benefit only LBC was clearly unethical, and impractical). The study hypothesis is that a community-based intervention can improve psychosocial well-being and academic performance of LBC, and rural children living with parents. The secondary aims were: (1) to compare baseline sociodemographic and psychosocial well-being measures between LBC and RC, (2) to conduct a community-based intervention, aimed at improving psychosocial well-being of LBC and RC, and (3) to assess the impact, sustainability and replicability of the intervention.

## 2. Materials and Methods

### 2.1. Setting and Participants

**Figure 1:**
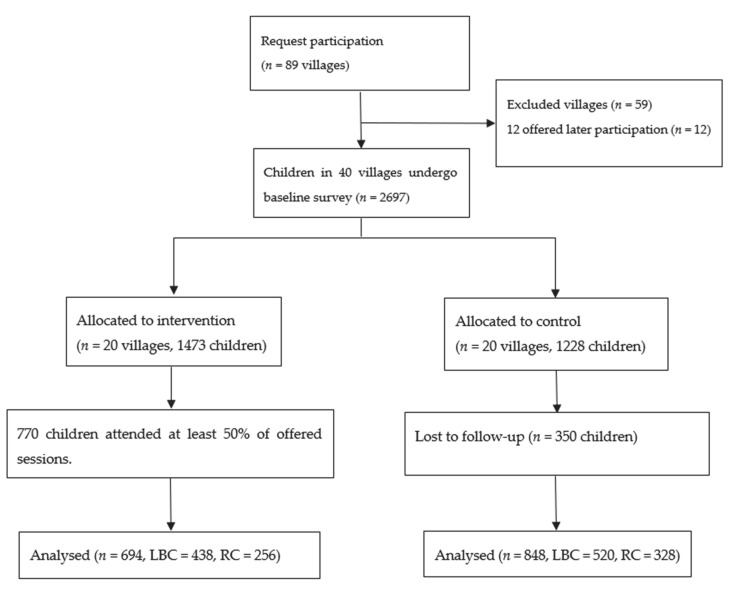
Study flow diagram.

To conduct this project we partnered with the Chinese Women’s Federation, a government organization with representation at all levels of Chinese society, which takes responsibility for aspects of the welfare of women and children. The study was conducted in Zhejiang Province, a relatively wealthy eastern province with a GDP per capita ranked third of all Chinese provinces. Our study sites were villages in three counties, all typical migrant-sending areas, across the economic spectrum, in the poorest prefecture of Zhejiang Province. A recent (unpublished) study showed that 59% of all children in rural Zhejiang were classified as left-behind, with 60% of these left-behind by both parents. Around 90% were in the care of grandparents, and 5% lived alone [21]. The intervention was conducted at the village level. The Women’s Federation informed village leaders across the three counties about the planned intervention, and 89 expressed interest in involvement; 32 villages did not meet inclusion criteria, five withdrew, and 12 were offered delayed participation. Twenty villages were allocated to intervention, and 20 to control, based on matching for population size and economic status, to maximize similarity between intervention and control villages. Control villages were offered the opportunity for involvement at a later stage. See the flowchart in Figure 1.

For the purposes of this study we defined left-behind children, as children who were currently left behind and had been separated from one or both of their parents for at least one year. We have found this definition provides more clarity for children completing questionnaires than the six-month standard definition.

### 2.2. The Intervention

The intervention comprised a network of Children’s Centres, located in villages. While originally planned to target LBC aged 7 to 15, it was agreed that all children be welcomed, though those outside the age range were not included in the analysis. The community-based approach aimed to foster local ownership, and to allow flexibility to adapt project guidelines to suit local conditions and preferences. Because of the range of interpretations of the guidelines, this was classified as a quasiexperimental approach. The essential requirements were: (1) provision of a physical space for activities, (2) a local Women’s Federation representative to take overall responsibility, and (3) volunteers to manage the Centre. Centres had to be open at least once per week during term time and three times per week during school holidays. Local volunteers were women residents with experience of working with children. They were trained by a child psychologist in the organisation of age-appropriate activities, child development, psychological health, and child safety. Start-up funds were provided to upgrade basic facilities for education, play, and sports equipment, and to provide per diems for volunteers. These funds were supplemented by donations of books, equipment and computers from partner schools in two cities. Centre activities included play, reading, art, help with homework, sports and physical activities. Left-behind children were specifically given practical help to communicate with migrant parents. There is strong evidence that regular, quality communication with parents improves the well-being of LBC [22,23]. Demands on volunteers were especially high during the long, hot summer holiday, so links were developed with the two local colleges, which provided student volunteers for 11 of the Centres. All volunteers were required to keep records of attendance, activities undertaken, and any problems. In the control villages children underwent baseline and follow-up testing; the community received feedback about the well-being of the children, and the offer of receiving the intervention at a later date.

### 2.3. Evaluation

Our community-based intervention explicitly encouraged different modes of implementation across the study sites to meet local need. There was wide variation in the ratios of volunteers to children, the types of facility and activity, and opening times. To understand the effects of the intervention therefore necessitated a more complex approach to evaluation. We therefore used a quantitative and qualitative evaluation: a questionnaire survey at baseline and follow-up to assess the impact of the intervention on psychosocial well-being, social support and academic performance of the children attending the Centres (compared with controls), and semistructured interviews with stakeholders focusing on impacts, acceptance and satisfaction, as well as lessons for sustainability.

The baseline and follow-up survey of children included detailed questions on left-behind status. Parental education referred to the highest education completed by either parent. Economic status was measured by household possession of certain items: air-conditioner, washing-machine, refrigerator, computer, and vehicle, with 4–5 scoring well-off, 2–3 fair, and 0–1 poor. Psychosocial well-being was assessed with the Chinese self-report version of the Strengths and Difficulties Questionnaire (SDQ-C) [24] This is validated and widely used in China. As recommended for general population samples, we used the three-subscale division of the SDQ, combining emotional and peer subscales into ‘internalizing problems’, (10 items), conduct and hyperactivity symptoms into externalizing problems (10 items) [25]. Except for the prosocial scale (five items) higher scores indicate greater difficulties. The Cronbach α for total difficulties was 0.79, with 0.73 and 0.84 for internalizing and externalizing problems, respectively. Social support was measured with the Chinese version of the Multidimensional Scale of Perceived Social Support (children’s version), which is also validated for China [26,27]. A score of 4 was categorized as high social support, 2–3 medium, and 0–1 low. School performance was measured by self-report with options of low, middle and high, scored as 1 to 3 respectively. Students in China are all informed of their class ranking. At follow-up, children were asked about attendance at the Centres, the frequency, and if they did not attend, why not.

### 2.4. Data Collection 

Survey data: In the 40 villages all children aged between 7 and 15 were invited to participate in the baseline survey. Questionnaires were administered by research assistants face-to-face, or by self-completion depending on the age, literacy, and preference of the child. The postintervention questionnaire was administered at a minimum of one year (range 13 to 15 months) after the start of the intervention.

Qualitative data: This was conducted by independent evaluators, and not members of the research team, in order to minimize reporting bias. In a random subsample of six intervention villages, semistructured interviews were conducted with 25 children, 18 parents, 18 carers, 16 volunteers, and 10 village leaders. Focus was on the acceptability, feasibility, and sustainability of the Centres, issues with implementation, and the community impact. All interviews were audio-taped and transcribed verbatim with specific consent from the participants.

The baseline survey was conducted in September to November 2015, the village interventions started from February to June 2016, and the postintervention assessment from September 2017 to January 2018.

Ethical approvals were obtained from University College London Research Ethics Committee 1654/005 and the Ethics Committee of Zhejiang University (Project Number ZJU 20141202). Consent from parents and/or carers, and assent from all children participating in the Centres was obtained. Great care was taken to ensure that children were treated with utmost sensitivity, given the potential vulnerability of many of these children.

### 2.5. Analysis

Quantitative analyses compared children in intervention villages, who had attended at least 50% of the sessions offered by their Centre, with children in control villages, who had no exposure to the intervention. For the SDQ to identify and compare proportions of children with problems, we used the recommended cut-off points for raised levels of total difficulties, as well as for internalizing and externalizing problems for Chinese populations: 14, 8 and 6 points respectively [25]. Mann—Whitney U tests compared changes in school performance and social support. Multivariate analyses were adjusted for age, sex, and parental education and family economic status. Baseline values were included in the models as covariates. All interview data were analysed thematically and are presented by interviewee group in summary form.

## 3. Results

### 3.1. Baseline Characteristics

These are shown in Table 1. Around 90% of the children residents aged 7 to 15 completed questionnaires: 1473 in intervention villages, (including 55.7% LBC), and 1224 in control villages, (including 58.4% LBC). Children in intervention and control villages were well-matched in terms of socio-demographic and well-being characteristics at baseline.

### 3.2. Impact of the Intervention on Psychosocial Well-Being

A total of 920 children were recorded as ever-attending the Centres, with a mean of 52 registered at each Centre (range 27 to 62). Of these, 770 attended at least 50% of the sessions and were thus eligible for inclusion in the analysis. Of these we were able to follow-up 694 children, 438 LBC and 256 RC, a response rate of 90%. The reasons given for children not attending the Centres were: (1) they didn’t like them (*n* = 36), (2) they weren’t allowed to (*n* = 20), or they were too far from their home (*n* = 7).

Differences between baseline and follow-up for SDQ measures, compared with controls, (for children left behind by both parents, for children left behind by one parent, and rural children) are shown in Table 2. After adjustment for age, sex, parental education, and family economic status, the intervention resulted in significant, but small, improvements in total difficulties in children left behind by both parents (OR =0.57, 95% CI 0.36–0.82 *p* = 0.009), children left behind by one parent (OR =0.62, 95% CI 0.39–0.84 *p* = 0.008 and RC (OR= 0.64 95% CI 0.29–0.98, *p* = 0.05) compared with controls. Changes in internalizing problems contributed most to the overall improvement, for both categories of LBC *p* < 0.001 and RC *p* = 0.006. There were no significant changes in externalizing problems in any of the groups.

As shown in Table 3, the post-intervention mean school performance significantly improved in both categories of LBC (*p* < 0.001), but not in RC (*p* = 0.07) and the social support score increased in both categories of LBC (*p* < 0.001, as well as RC (*p* = 0.01).

### 3.3. Summary of Interview Findings 1

Children were unanimously positive about the Centres, looking forward to attending rather than being “stuck at home” with grandparents. Many described feeling happier since the Centres had opened, and many wanted them to open for longer hours. Children developed strong bonds with volunteers, who they turned to for help with problems. Several children said they felt closer to volunteers than their parents, and student volunteers were especially popular, particularly, as noted by several children, for their ability to help with homework.

Migrant parents were very supportive of the clubs, talking of feeling reassured that the children were attending, that the clubs provided a place of safety, help with homework, and were free of charge. However, a number of parents thought the Centres might actually encourage parents to migrate, because the pressure on grandparents is relieved, and children are safe and occupied outside school hours, so parents feel less guilt about separation from their children. Around half the parents said they would be willing to pay an attendance fee, if it helped to ensure the Centres remained open.

Grandparent carers were mostly supportive, with issues of safety and help with homework mentioned most frequently. Some also valued the Centres as a place to socialise with other carers. Several complained that attending the Centres meant children had less time for household chores.

The interviews revealed that local volunteers were divided fairly equally between: (1) those who loved the work and responsibility, gained satisfaction from it, and for whom the Centres had become an important part of their lives, and (2) those who had found volunteering to be challenging and difficult. Two said they felt their hard work was not appreciated by the community. (Both of these resigned soon after these interviews took place). Most of the volunteers wanted to be paid more than the per diem provided. Several said there should be a charge for attendance, to improve the chances of the Centres’ sustainability.

The views of the village leaders were mixed. Some expressed pride in their Centre, with frequent mention of the Centres benefiting the whole community, and describing the Centre as an important “community asset”. Village leaders talked of a competitive spirit created by intervillage rivalry for the best Centre. Most expressed awareness of the importance of strong leadership for the successful running of the Centres. The leadership qualities specified included enthusiasm, proactive management, prompt trouble-shooting of problems, and overall support. There were also concerns about the additional work and responsibility it entailed. One village leader talked about the limitations of the “volunteer spirit”, and the danger of relying on a few dedicated volunteers. All praised the dedication of the volunteers, without whom the Centres would not exist. Two of the leaders in villages where student volunteers were not involved, said they would like to be able to access student volunteers, and that this would be necessary to ensure sustainability. Two village leaders had concerns about the most vulnerable children, the poorest, the disabled, and children living in isolated situations outside villages, who were least able to attend, but might benefit most. Finally, concerns were expressed about sustainability, and the need for long-term government funds, or a charge for attendance, to make this possible.

## 4. Discussion

Our study provides evidence that a community-based intervention, consisting of Children’s Centres, can improve aspects of psychosocial well-being, including academic performance, in both LBC and RC in rural Zhejiang Province. LBC benefited slightly more than RC, especially regarding internalising problems and school performance. Importantly, the intervention demonstrated a positive impact on most of the communities as a whole.

The improvement, albeit small, in self-reported school performance, especially among LBC, is of particular importance in the Chinese setting where academic achievement is highly prized, and is viewed as a route out of poverty [28]. Many of the parents, carers and children talked of the importance of the Centres as places where homework could be supervised, and they attributed improvements in school performance to this. The inability of grandparents to supervise or help with homework, largely because of their own lack of educational opportunity, has been cited as one of the reasons why LBC fall behind at school in some parts of rural China [29].

The very few other published studies about interventions for LBC have been small scale and targeted: a family workshop intervention for preschool children [13], two studies involving psychological counselling [15,16], and a study involving group play [14]. There are two published community-based studies: one from Mexico and one from China. The latter shows how a programme of support called the Children’s Companion Mother Programme has improved resilience, physical health and academic performance in LBC [17]. Our findings concur with the conclusions drawn by the authors of that study, that the vulnerabilities and difficulties of LBC caused by the lack of parental presence, can be mitigated through a strong supportive social structure in the local community.

However, while our study shows some small improvements in aspects of well-being in LBC and rural children, the key lessons from the study relate to aspects of the methodological approach. We encouraged communities to adopt an intervention to suit local conditions and needs, while complying with broad project guidelines. There is growing interest in the importance and value of the adaptation of trial design to the “real world” as a way of better informing evidence-based policy and practice. In addition, the use of mixed-methods evaluation, particularly qualitative approaches, leads to better understanding of the process and outcomes of the intervention, with clear implications for dissemination and replication [30]. We expand on these approaches below.

Firstly, using a quasiexperimental trial design, we allowed villages to adapt the project to meet local needs and preferences. So the trial comprised a range of somewhat differing local interventions, which is not standard in the scientific literature. But the approach proved very successful in increasing the sense of local ownership, responsibility and pride, and even created intervillage rivalry for the “best” Centre. Adhering to a strict trial design, without the option of adaptation to the “real world” situation, would not have yielded such positive results.

Secondly, we demonstrate the importance of mixed methods in evaluation of interventions. None of the other published interventions focussing on LBC used qualitative methods for evaluation [13,14,15,16,17,18]. We found our qualitative measures to be crucial to our understanding of both process and outcomes. While the quantitative findings demonstrated some significant, but small, improvements in a few psychosocial measures, the qualitative findings provided different but important types of evidence: (1) many children described really enjoying attending the Centres and they looked forward to going to them, and this made them feel happier, an attribute which is difficult to measure quantitatively because of its subjectivity, (2) positive evaluation of the Centres, especially by children and parents, (3) the range of implementation types, and which elements contributed to success, (4) the wider effects of the intervention, for example, peace of mind for absent parents, respite care and increased social interaction for grandparent carers, and the benefits for volunteers, many of whom thrived in the role. Without the qualitative component, these findings would not have emerged. This demonstrates that evaluation using mixed methods provides a more nuanced approach, valuable to policy guidance [30].

The lessons from this intervention have informed policy. The intervention was able to leverage the political will generated by the Chinese government’s 2013 call to prioritise the care and education of left-behind children [11]. This announcement was repeated in 2016 to include children in difficult circumstances and not just LBC. Responsibility for delivering on these calls lies with local communities. Our study highlighted the key factors which are crucial to successful implementation of a project to support vulnerable children at low cost, providing lessons for elsewhere. The major lessons are: local political will, strong leadership, support of the community, and motivated, enthusiastic volunteers. Through the Women’s Federation national network the lessons have been widely disseminated. We are aware that the model has been adopted in migrant-sending areas of at least two other provinces.

We have also adapted the model to continue the project in eight villages in Zhejiang. Finding suitable local volunteers proved a major challenge in around half the original project villages. But there was no shortage of enthusiastic student volunteers. In China students are required to participate in social responsibility activities, as part of their curriculum, and projects of this type fit that remit. With the Women’s Federation, we are now working with a local higher education college to continue the Centres in six villages. A core of student volunteers run the Centres at weekends and in the holidays, under the supervision of the Women’s Federation representative. The colleges cover the travel costs of the students, and village families provide accommodation when needed. Importantly, efforts are made to attract (and physically collect if necessary) the most vulnerable children, the poorest, the disabled, and children living in isolated situations outside villages. The local government is providing a small amount of funding to ensure the Centres remain free of charge at present.

There are several limitations. First, the 40 villages were self-selected, and inevitably comprised those with good leadership, enthusiasm and capacity to develop a Centre. Second, the children who chose to attend the Centres and were followed-up were probably more motivated than those who did not attend, potentially biasing outcomes. Third, the study was conducted in Zhejiang, a relatively wealthy province, where some local financial support for the project was available. This raises questions about replicability in poorer areas of China. Fourth, reliance on self-report may have resulted in social desirability bias, though the qualitative research was conducted by independent evaluators who explicitly encouraged open and honest responses. Finally, this was planned as a one-year, intensive programme, but it may not have been long enough for changes in outcomes to become manifest. The six villages where the project is ongoing may provide the opportunity for longer-term and more detailed follow-up.

## 5. Conclusions

The model demonstrates that with strong leadership and community motivation, it is possible to carry-out a low-cost and beneficial intervention to improve psychosocial well-being in LBC and rural children. Our findings suggest that such Centres not only represent a potential investment for the future of these children, but also contribute to community cohesion and well-being. The model may inform interventions to support children in other migrant-sending communities, but is most appropriate for rural China, where the sociocultural contexts are similar.

## Figures and Tables

**Table 1:** Baseline characteristics of children in intervention and control villages.

		Intervention Villages	Control Villages	*p*-Value
		(*n* = 1473)	(*n* = 1224)	
Sex	Male	745 (50.6)	624 (51.0)	
	Female	728 (49.4)	604 (49.3)	0.68
Left-behind status	Left behind both parents	497 (33.7)	399 (32.6)	
	Left behind one parent	323 (21.9)	318 (25.9)	
	Rural	653 (44.3)	507 (41.4)	0.15
Age	Mean (SD)	11.8 (2.31)	11.6 (2.24)	0.34
Family economic status	Poor	390 (26.4)	288 (23.5)	
	Moderate	675 (45.8)	585 (47.7)	
	Well-off	408 (27.6)	351 (28.6)	0.09
Parents education	Primary school	462 (31.3)	343 (28.0)	
	Middle school	677 (45.9)	575 (46.9)	
	High school	265 (17.9)	228 (18.6)	
	College	69 (4.7)	78 (6.1)	0.12
Primary carer	Grandparent	666 (46.3)	575 (46.9)	
	Father	173 (12.0)	126 (10.3)	
	Mother	531 (36.9)	479 (39.1)	
	Other	67 (4.6)	48 (3.9)	0.09
Parents marital status	Parents married	1231 (85.6)	1064 (86.9)	
	Divorced/separated	159 (11.1)	115 (9.4)	
	Other	83 (5.7)	45 (3.6)	0.06
School performance	Very poor/poor	403 (28.0)	271 (22.1)	
	Average	605 (42.1)	559 (45.6)	
	Very good/good	429 (29.8)	394 (32.1)	0.08
Social support	Low	232 (15.7)	159 (12.9)	
	Average	639 (43.4)	597 (48.7)	
	High	602 (40.8)	468 (38.2)	0.03
SDQ scores	Total difficulties	12.9 (5.1)	12.8 (4∙8)	0.08
	Internalising problems	6.6 (3.7)	6.7 (3.9)	
	Externalising problems	6.3 (3.1)	6.1 (2.8)	
	Prosocial behaviour	7.3 (3.2)	7.5 (3.0)	0.42

**Table 2:** Baseline and end-of-study assessments of children in intervention villages and children in control villages by left-behind status—Strengths and Difficulties Questionnaire (SDQ)

	Baseline %	End of Study %	Change %	Odds Ratio for Intervention vs. Control	*p*-Value
**Total Difficulties Score > 14 (%)**					
Intervention LBC—both parents (*n* = 267)	41.2	31.9	−9.3	0.57 (0.36, 0.82)	0.009
Control LBC—both parents (*n* = 302)	37.5	37.9	+0.4		
Intervention LBC—one parent (*n* = 171)	43.7	33.9	−9.8	0.62 (0.39,0.84)	0.008
Control LBC—one parent (*n* = 218)	35.2	34.1	−1.1		
Intervention RC (*n* = 256)	46.3	38.7	−7.6	0.64 (0.29, 0.98)	0.05
Control RC (*n* = 328)	44.3	43.1	−1.2		
**Internalizing Problems Score > 6 (%)**					
Intervention LBC—both parents	56.4	45.4	−11.0	0.42 (0.21,0.71)	<0.001
Control LBC—both parents	55.6	53.2	−2.4		
Intervention LBC—one parent	51.8	41.2	−10.6	0.39 (0.23,0.69)	<0.001
Control LBC—one parent	49.2	40.1	−9.1		
Intervention RC	52.2	44.5	−7.7	0.55 (0.35,0.82)	0.006
Control RC	50.6	52.8	+2.2		
**Externalizing Problems Score > 8 (%)**					
Intervention LBC—both parents absent	20.5	17.4	−3.1	0.69 (0.33,1.42)	0.31
Control LBC—both parents absent	20.9	23.1	+2.2		
Intervention LBC—one parent absent	19.4	18.9	−0.5	0.82 (0.55.1.51)	0.45
Control LBC—one parent absent	18.6	18.7	+0.2		
Intervention RC	20.5	17.4	−3.1	0.84 (0.58, 1.21)	0.92
Control RC	27.6	27.4	−0.2		
**Prosocial Score < 7 (%)**					
Intervention LBC—both parents absent	40.5	32.2	−8.3	0.72 (0.55,0.99)	0.05
Control LBC—both parents absent	34.7	35.5	+0.8		
Intervention LBC—one parent absent	42.8	35.8	−7.0	0.77 (0.61,1.2)	0.07
Control LBC—one parent absent	38.5	34.5	−4.0		
Intervention RC	45.5	40.8	−4.7	0.83 (0.62, 1.21)	0.8
Control RC	42.4	42.9	+0.5		

**Table 3:** Baseline and end-of-study assessments, mean school performance and social support, of children in intervention villages and control villages by left-behind status.

		Baseline	End of Study	*p*-Value
Mean school performance (SD)				
LBC—both parents absent	Intervention	2.02 (0.4)	2.18 (0.5)	<0.001
	Control	2.05 (0.3)	2.04 (0.3)	
LBC—one parent absent	Intervention	2.04 (0.2)	2.20 (0.4)	<0.001
	Control	2.06 (0.4)	2.08 (0.4)	
Rural Children	Intervention	2.10 (0.4)	2.15 (0.5)	0.07
	Control	2.00 (0.2)	2.01 (0.2)	
Mean social support (SD)				
LBC—both parents absent	Intervention	3.23 (1.8)	3.66(2.0)	<0.001
	Control	3.08 (1.3)	3.10 (1.4)	
LBC—one parent absent	Intervention	3.31(1.2)	3.52 (2.1)	<0.001
	Control	3.35 (1.3)	3.39 (1.5)	
Rural Children	Intervention	3.15 (1.5)	3.32 (0.7)	0.005
	Control	3.11 (1.1)	2.98 (1.1)	
